# PGC-1α regulates critical period onset/closure, mediating cortical plasticity

**DOI:** 10.3389/fnmol.2023.1149906

**Published:** 2023-09-25

**Authors:** Wei-Jun Zhang, Hou-Zhen Shi, Mei-Na Guo, Long-Fei Xu, Hong-Ru Zhai, Zi-Zhong Liu, Yong-Qiang Zhu, Wei-Ning Zhang, Jia Wang

**Affiliations:** ^1^The Fourth Affiliated Hospital of Jiangsu University, Zhenjiang, Jiangsu, China; ^2^Department of Laboratory Medicine, School of Medicine, Jiangsu University, Zhenjiang, Jiangsu, China; ^3^Zhenjiang Jieshengrui Biotechnology Co., Ltd., Zhenjiang, Jiangsu, China

**Keywords:** cortex, matrix metalloproteinase 9 (MMP9), parvalbumin interneurons (PVIs), perineuronal nets (PNNs), PGC-1alpha

## Abstract

Peroxisome proliferator-activated receptor PPARγ coactivator-α (PGC-1α) is concentrated in inhibitory interneurons and plays a vital role in neuropsychiatric diseases. We previously reported some characteristic features of schizophrenia (SZ) in GABAergic neuron-specific *Pgc-1alpha* knockout (KO) mice (Dlx5/6-Cre: *Pgc*−1*alpha*^f/f^). However, there is a fundamental gap in the molecular mechanism by which the *Pgc-1alpha* gene is involved in the neurobehavioral abnormalities of SZ. The loss of critical period (CP) triggers–maturations of parvalbumin interneurons (PVIs) and brakes—and the formation of perineuronal nets (PNNs) implicates mistimed trajectories during adult brain development. In this study, using the *Pgc-1alpha* KO mouse line, we investigated the association of *Pgc-1alpha* gene deletion with SZ-like behavioral deficits, PVI maturation, PNN integrity and synaptic ultrastructure. These findings suggest that *Pgc-1alpha* gene deletion resulted in a failure of CP onset and closure, thereby prolonging cortical plasticity timing. To determine whether the manipulation of the PNN structure is a potential method of altering neuronal plasticity, GM6001, a broad-spectrum matrix metalloproteinase (MMP)-inhibitor was applied. Here we confirmed that the treatment could effectively correct the CP plasticity window and ameliorate the synaptic ultrastructure in the *Pgc-1alpha* KO brain. Moreover, the intervention effect on neuronal plasticity was followed by the rescue of short-term habituation deficits and the mitigation of aberrant salience, which are some characteristic features of SZ. Taken collectively, these findings suggest that the role of PGC-1α in regulating cortical plasticity is mediated, at least partially, through the regulation of CP onset/closure. Strategically introduced reinforcement of molecular brakes may be a novel preventive therapy for psychiatric disorders associated with PGC-1α dysregulation.

## Introduction

Locally projecting interneurons that release inhibitory neurotransmitter γ-aminobutyric acid (GABA) are arguably the most diverse cell population in the brain (Markram et al., 2004). A few interneuron subsets are preferentially affected in developmental (Di Cristo, 2007) and psychiatric disorders, such as schizophrenia (SZ) (Lewis and Gonzalez-Burgos, 2008). Only a limited number of transcriptional cues by which specific interneuron subsets mature and function during brain development have been confirmed (Lucas et al., 2010). The increasing emphasis on the neuronal pathology of SZ has led to the identification of abnormalities in parvalbumin interneurons (PVIs) in some key brain regions, including the hippocampus and the cortex (Patrono et al., 2023).

Parvalbumin (PV) is one of three Ca^2+^-binding proteins that, together with calbindin and calretinin, are expressed in largely non-overlapping populations of GABAergic neurons. In recent years, the PV-containing subclass of interneurons has been found to be particularly instrumental in synchronizing the action potential generation of entire networks of principal cells into characteristic patterns of activity, known as gamma oscillations (Antonoudiou et al., 2020). Gamma oscillations are suspected to play a vital role in recognition and memory and are abnormal in SZ. Accordingly, decreased PV expression is a hallmark feature in the neuropathology of SZ (Enwright et al., 2016). It is worth noting that PV-positive GABA circuits are also involved in the regulation of critical period (CP) during brain development (Cisneros-Franco and de Villers-Sidani, 2019).

A number of processes within the central nervous system, including basic affective, intellectual, and social cognitions are “plastic;” this characteristic of plasticity normally takes shape during various CPs (Do et al., 2015) and is the strongest in children and young animals, which is also called developmental CP plasticity. A focus on the cellular and molecular bases of these developmental trajectories has begun to unravel the mechanisms that control the onset and closure of such CPs.

**Figure 1:**
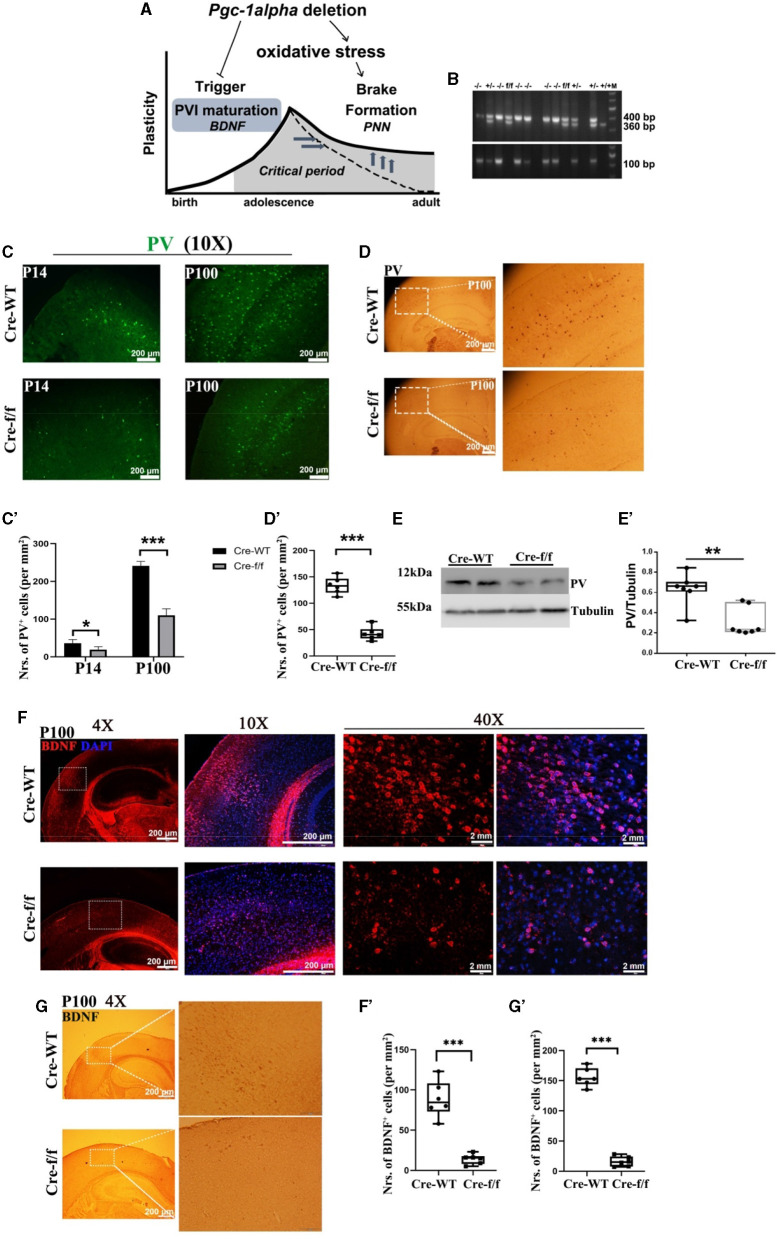
*Pgc-1alpha* deletion prolongs critical period plasticity due to a failure of PVI maturation. **(A)** *Pgc-1alpha* deletion prolongs critical period plasticity due to a failure of the onset of the CP window. **(B)** PCR products obtained from genomic DNA from Dlx5/6-Cre: *Pgc*−1*alpha*^fl/fl^ (–/–), Dlx5/6-Cre: *Pgc*−1*alpha*^fl/+^ (+/–), Dlx5/6-Cre: *Pgc*−1*alpha*^+/+^ (+/+), and *Pgc*−1*alpha*^fl/fl^ (f/f) mice. The 400- and 360-bp bands result from the amplification of *Pgc*−1*alpha*^fl/fl^ and wild-type alleles, respectively. The 100-bp band results from the amplification of the Dlx5/6-Cre allele. **(C)** Immunofluorescence using an antibody specific to parvalbumin (PV) was conducted on 30-μm coronal brain sections of *Pgc-1alpha*–/– (Cre-f/f) and *Pgc-1alpha*+/+ (Cre-WT) mice. Green, PV. Scale bars = 200 μm. **(C')** The number of PV^+^ cells from the cortex of Cre-WT and Cre-f/f mice are illustrated as the mean values obtained on days 14 and 100 during the postnatal period. Values are expressed as means ± S. E. M. *n* = 6/group. **(D)** Immunohistochemistry using an antibody specific to PV was conducted on 30-μm coronal brain sections of Cre-f/f and Cre-WT mice. Scale bars = 200 μm. **(D')** The quantification of PV for immunohistochemistry in the cortex is shown in the bar graph. Values are expressed as means ± S. E. M. *n* = 6/group. **(E–E')** Cortical lyases of *Pgc-1alpha* genotype mice were immunoblotted using an antibody against PV. The expression pattern and quantification of PV were studied by western blotting. Values are expressed as means ± S.E.M. For each group, *n* = 7. Tubulin was used as the loading control. **(F)** Micrographs show double-labeling for brain-derived neurotrophic factor (BDNF) and DAPI in the cortex of Cre-f/f and Cre-WT mice. Red, BDNF; Purple, DAPI. **(F')** The quantification of BDNF for immunofluorescence in the cortex is shown in the bar graph. **(G)** Immunohistochemistry using an antibody specific to BDNF was conducted on 30-μm coronal brain sections of Cre-f/f and Cre-WT mice. Scale bars = 200 μm. **('G')** The quantification of BDNF for immunohistochemistry in the cortex is shown in the bar graph. Values are expressed as means ± S. E. M. *n* = 6/group. Significant levels were set at **p < 0.01, ***p < 0.001 denotes noted differences between Cre-WT and Cre-f/f animals.

Excitatory-inhibitory (E-I) circuit balance is a trigger. Specific GABA circuit maturation underlies the onset timing of plasticity (Figure 1A). Among the diverse inhibitory cell types, the PV basket cell serves as the pivotal plasticity switch. PVI networks are interconnected via gap junctions and reciprocal GABAergic synapses and are capable of synchronizing the excitatory state of large numbers of pyramidal neurons (Do et al., 2015). PV-positive cells can adapt their intrinsic properties (cellular plasticity) and output (synaptic plasticity) in response to sensory experience (Favuzzi et al., 2017). PVIs mature at different rates across brain regions, contributing to the sequential timing of CP. They depend on a variety of extrinsic factors for their maintenance. Brain-derived neurotrophic factor (BDNF), one of the extrinsic factors that benefit the maintenance and health of PVIs, appears ahead of CP onset.

**Figure 2:**
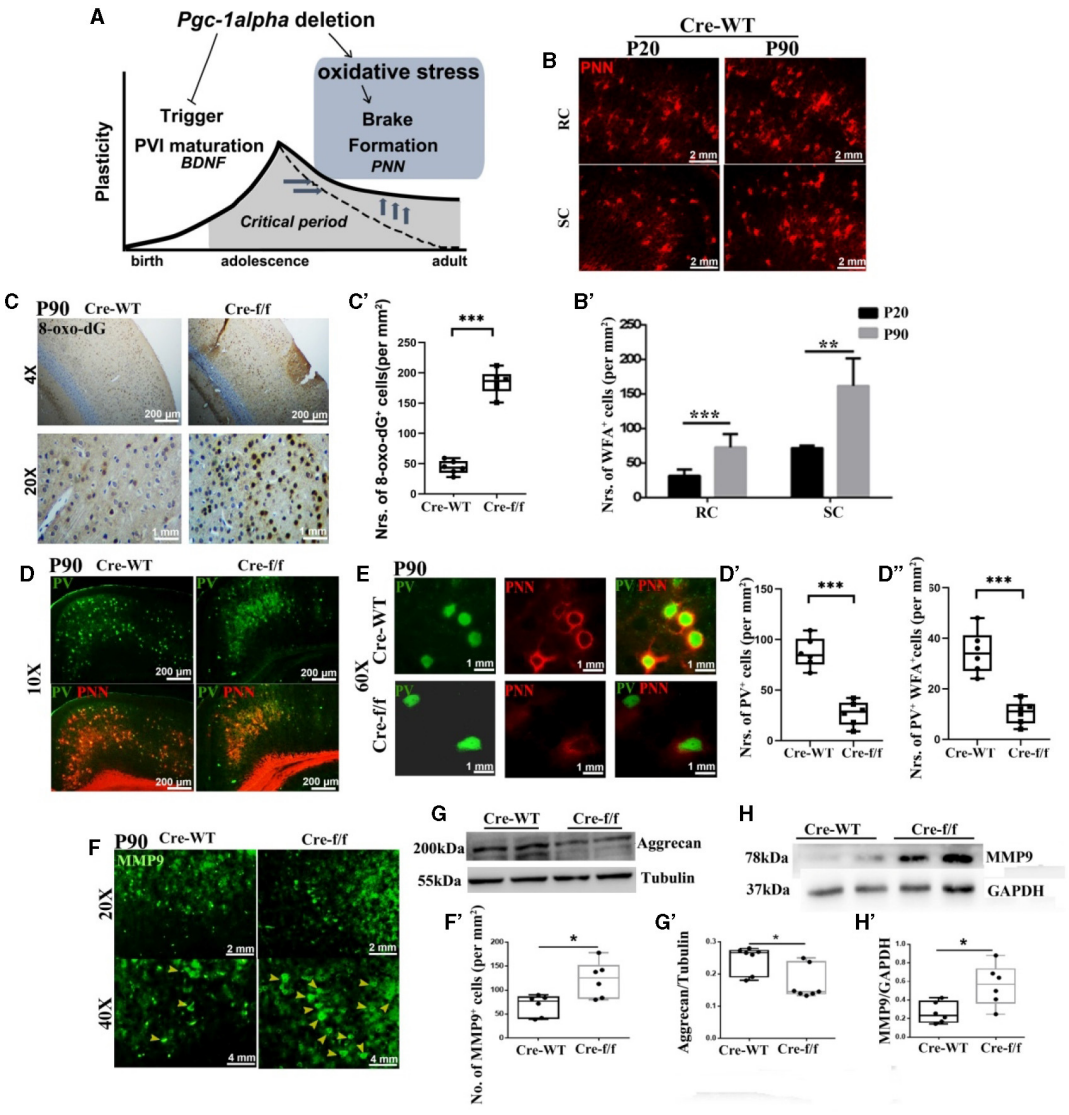
*Pgc-1alpha* deletion disturbs the closure of critical plasticity due to the deficits of PVI and their surrounding PNNs. **(A)** *Pgc-1alpha* deletion prolongs critical period plasticity due to a failure of the CP window closure. **(B)** Immunofluorescence using an antibody specific to *Wisteria floribunda* agglutinin (WFA)-labeled PNNs was conducted on 30-μm coronal brain sections of Cre-WT mice at the ages of P20 and P90. Red, WFA-labeled PNNs. Scale bars = 2 mm. **(B')** The number of WFA^+^ cells from the retrosplenial cortex (RC) and the somatosensory cortex (SC) of Cre-WT mice are illustrated as the mean values obtained on days 20 and 90 during the postnatal period. Values are expressed as means ± S.E.M. For each group, *n* = 6–7. **(C)** Immunohistochemistry using an antibody specific to 8-oxo-7,8-dihydro-20-deoxyguanine (8-oxo-dG) was conducted on 30-μm coronal brain sections of Cre-f/f and Cre-WT mice. **(C')** The quantification of 8-oxo-dG^+^ cells for immunohistochemistry in the cortex is shown in the bar graph. Values are expressed as means ± S.E.M. For each group, *n* = 6–7. **(D, E)** Micrographs show double-labeling for PV and WFA-labeled PNNs in the cortex of Cre-f/f and Cre-WT mice. Green, PV; Red, WFA-labeled PNNs. **(D'–D”)** The number of PV^+^ cells and PV^+^WFA^+^ cells obtained from the cortex of Cre-WT and Cre-f/f mice are illustrated as the mean values obtained on day 90 during the postnatal period. Values are expressed as means ± S.E.M. For each group, *n* = 6–7. **(F)** Immunofluorescence using an antibody specific to matrix metalloproteinase 9 (MMP9) was conducted on 30-μm coronal brain sections of Cre-f/f and Cre-WT mice. Green, MMP9. Arrowhead: MMP9^+^ cell. **(F')** The number of MMP9^+^ cells obtained from the cortex of Cre-WT and Cre-f/f mice are illustrated as the mean values obtained on day 90 during the postnatal period. Cortical lyases of *Pgc-1alpha* genotype mice were immunoblotted using antibodies against Aggrecan and MMP9. The expression pattern and quantification of Aggrecan **(G–G')** and MMP9 **(H–H')** were studied by western blotting. Values are expressed as means ± S.E.M. For each group, *n* = 7. Tubulin/GAPDH was used as the loading control. Significant levels were set at *p < 0.05, and ***p < 0.001 denotes noted differences between Cre-WT and Cre-f/f animals.

A previous study reported that CP closure is marked by the condensation of perineuronal nets (PNNs) (Figure 2A). As PVIs mature, they gradually acquire an extracellular coating called PNNs, which limit excessive circuit rewiring in adulthood (Takesian and Hensch, 2013). Molecular “brakes” limit adult plasticity to stabilize neural networks. The loss of PNNs in adult brains is consistent with a delayed or extended period of instability (Wen et al., 2018b). Correspondingly, various neural processes that should be consolidated during brain development seem to remain open to fluctuations in adult patients (Do et al., 2015). A number of studies based on post-mortem brain tissues of patients with SZ have demonstrated that there is a disease-specific reduction in the density of PNNs, as well as the altered expression of genes that regulate PNNs in key brain structures associated with psychiatric disorders (Kim et al., 2017).

According to these reports, blocking PNN damage through pharmacological or genetic means could present a therapeutic strategy for psychiatric disorders. Recent findings have revealed that matrix metalloproteinase 9 (MMP9) targeting the PNN component precisely regulates molecular and cellular modification in PVIs (Dwir et al., 2020). The above findings, along with the finding that aggrecan, which is an important component of PNNs, is a direct target of MMP9 (Madsen et al., 2010), have tempted us to explore the key regulators that can orchestrate MMP9-mediated pruning of PNNs, thereby warranting and potentially paving the way for novel therapeutic treatment strategies for neurological disorders.

The transcriptional coactivator peroxisome proliferator-activated receptor 1 alpha (PGC-1α) has been coined the “master regulator of metabolism” owing to its ability to induce gene programs that control mitochondrial biogenesis and antioxidant production (Rius-Perez et al., 2020; Wang et al., 2022a). A previous observation has confirmed that PGC-1α could regulate the gene program, primarily in GABAergic neurons (Cowell et al., 2007). Consistent with this finding, we previously generated *Pgc-1alpha* conditional knockout mice through the transgenic expression of Cre recombinase under the control of the dlx5/6 promoter, resulting in Cre-mediated excision events occurring specifically in GABAergic neurons (Wang et al., 2019). Using the specific mouse line, we reported some characteristic features of SZ in GABAergic neuron-specific *Pgc-1alpha* knockout mice (Dlx5/6-Cre: *Pgc*−1*alpha*^fl/fl^) (Wang et al., 2020a). These behavioral dysfunctions were associated with decreased PV expression in the cortex and hippocampus (Wang et al., 2020b). These, along with the description that the loss of PVIs and PNNs are implicated in mistimed brain development trajectories (Do et al., 2015), have tempted us to pose two questions: (1) Does PGC-1α regulate the CP plasticity window during adult postnatal development? and (2) Can we target the PNN-mediated closure of CP timing and thereby ameliorate cortical plasticity in adult *Pgc-1alpha* KO mice? To answer these questions, using the *Pgc-1alpha* KO mouse line, in this study, we investigated the association between *Pgc-1alpha* deletion and the maturation of PVI and their surrounding PNNs, MMP9 expression, and synaptic ultrastructure; we also probed the intervention effect of an MMP inhibitor on SZ-like behavioral manifestations, as well as its potential mechanism, with a particular focus on cortical plasticity.

## Materials and methods

### Animals

*Pgc*−1*alpha*^fl/fl^ mice were obtained from the Jackson Laboratory (Jax mouse Cat No. 009666). Dlx5/6-Cre: *Pgc*−1*alpha*^fl/fl^ (Cre-f/f) mice (Figure 1B) were generated by crossing mice expressing the *Pgc-1alpha* gene flanked by loxP sites (*Pgc-1alpha* floxed) with mice expressing Cre recombinase in GABAergic interneurons (CJ Zhao, Medical School of Southeast University, China) (Wang et al., 2019, 2020a). Dlx5/6-Cre: *Pgc*−1*alpha*^+/+^ (Cre-WT) mice were used as the control. The experiments were conducted in accordance with the policies established by Jiangsu University (SYXK2018-0053), the Chinese Council on Animal Care, and the National Institutes of Health Guide for the Care and Use of Laboratory Animals (NIH Publications No. 8023, revised 1978).

### Experimental design

To address whether *Pgc-1alpha* deletion affects the molecular components associated with brain development, the cortex of the Dlx5/6-Cre: *Pgc*−1*alpha*^+/+^ (Cre-WT) mice and Dlx5/6-Cre: *Pgc*−1*alpha*^fl/fl^ (Cre-f/f) mice at the age of P14 (infant) and P90-100 (adult) were investigated. The animals were housed in an animal vivarium under a reversed light–dark cycle (lights on: 1900–0700 h) and provided with food and water *ad libitum* throughout the entire study. Following drug treatment, the animals were caged individually and handled daily. All behavioral manipulations were conducted during the dark phase of the cycle.

### Drugs

GM6001 (Meilun, Dalian, China) was dissolved in dimethyl sulfoxide (DMSO) as a stock solution (200.79 mM) and further diluted in 10% polyoxyethylene sorbitan monolaurate (Tween 20) to a total volume of 400 μL before administration (final DMSO concentration: 20%). The GM6001 (100 mg/kg) and vehicle (400 μL, 20% DMSO, and 10% Tween 20 in saline) were administered intraperitoneally once a day for 11 days.

### Groups

Two independent cohorts of naïve male mice were employed for Experiment 1 (n = 16) and Experiment 2 (n = 16). In Experiment 1, two genotypes, namely, Cre-WT and Cre-f/f, were applied to evaluate the association between *Pgc-1alpha* gene deletion with and behavioral abnormalities and molecular mechanisms relevant to cortical plasticity. In Experiment 2, two treatments, namely, vehicle and GM6001, were applied to explore whether blocking the damage of PNNs could restore behavior manifestation and the synaptic plasticity window of *Pgc-1alpha* KO mice.

### Western blotting

The mice (*n* = 6-8 in each group) were decapitated, the entire brain was removed, and samples of the cortex were immediately dissected, frozen, and stored at−80°C. Subsequently, the samples were lysed, and Western blotting (WB) was performed, as previously described (Wang et al., 2019). The primary antibodies included rabbit anti-aggrecan antibody (1:500; Wanleibio, WL02316, Shenyang, China), rabbit anti-PV (1:500, Proteintech, 29312-AP, Chicago, IL, USA), rabbit anti-MMP9 (1:1,000, Wanleibio, WL03096, Shenyang, China), rabbit anti-postsynaptic density protein 95 (PSD95) (1:2,000, Beyotime, AG4750, Shanghai, China), rabbit anti-synaptophysin (SYP) (1:1,000, Beyotime, AF8091, Shanghai, China), rabbit anti-β-tubulin (1:2,000, Abcam, EPR16774, Vancouver, Canada), and rabbit anti-GAPDH (1:3,000, Abcam, EPR16891, Vancouver, Canada). In addition, goat anti-rabbit horseradish peroxidase-linked secondary antibodies (1:5,000, Beyotime, A0208, Shanghai, China) were used to probe the blots. Protein visualization was carried out by enhanced chemiluminescence (ECL, Beyotime, p00185, Shanghai, China). The signal intensity was obtained by densitometric scanning.

### Immunohistochemistry

The mice (*n* = 6-8 in each group) were anesthetized with ethyl carbamate; they were perfused transcardially with PBS and then with 4% paraformaldehyde (PFA) in 0.01 M PBS, pH 7.4 and then Brain tissues were removed and placed in 4 % PFA for 24 h. The brain tissues were then transferred to 30 % sucrose for 24 h. Subsequently, 30-μm-thick coronal frozen sections were used to investigate the outer two-thirds of the cortex, and immunohistochemistry (IH) was performed, as previously described (Wang et al., 2022b). To visualize PNNs that specifically surround PVIs, sections were incubated in a solution containing lectin *Wisteria floribunda* agglutinin (WFA). The following antibodies and reagents were used for IH: biotin-conjugated WFA (1:500; Sigma, Shanghai, China), rabbit anti-MMP9 (1:1,000, Wanleibio, WL03096, Shenyang, China), mouse anti-PV (1:500, Proteintech, AG3042, Beijing, China), rabbit anti-BDNF (1:500, Proteintech, AF1423, Beijing, China), 8-oxo-7,8-dihydro-20-deoxyguanine (8-oxo-dG) (1:200, Bioss, bs-1278R, Beijing, China), Dylight 488 (cat # BA1126, Wuhan, China), CY3 (cat # BA1032, Wuhan, China), DAPI (KeyGen Biotech, cat # KGA215-50, Shanghai, China), HRP Vision IgG antibody (Boster, cat # SV0001, Wuhan, China), DAB (Bosterbio, AR1000, Pleasanton, CA, USA), and hematoxylin (Beyotime, C0107, Beijing, China).

### Scanning electron microscopy

A series of 50-μm-thick sections−250 μm apart from each other—were collected from the cortex (from Bregma, ~-2.9; lateral, ~±0.25; depth, ~-0.6 mm) of mice. After washing in PBS, the sections were treated with 0.5% osmium-tetroxide for 20 min, dehydrated, and embedded in epoxy resin. During dehydration, the sections were treated with 1% uranyl acetate. After polymerization, we prepared 70-nm-thick sections (Leica EM UC6, Wetzlar, Germany) of the outer two-thirds of the cortex, picked them up on formvar-coated single-slot copper grids, and examined them using a JEOL-1200EX electron microscope (EM) and a Soft Imaging System Veleta CCD camera (EMSIS, Münster, Germany). The synaptic area was measured from serial sections of three-dimensional (3D) reconstructed synapses. We only included synapses that were cut perpendicularly to the sectioning plane.

### Imaging and analyses

For cell counting, coronal cortical sections were examined in two to three comparative sections from the anterior to posterior levels in each brain by an experimenter blind to the group. The cells were counted in a minimum of three sections taken from each brain. To define the counting area, we used Image Pro Plus software (Media Cybernetics) to outline the areas of interest module.

For measurements of electron microscopy images, including the synaptic cleft width and the postsynaptic density (PSD) width, we used Image J software. Synaptic and postsynaptic grayscale values were investigated. The distance between the two highest local grayscale maxima represented the actual synaptic cleft width, and the distance between the highest postsynaptic local maximum and the first local minimum represented the PSD width. Counts were averaged per group (*n* = 6–8/group) and normalized by the volume of tissue investigated. For each animal, digital images from six brain sections were analyzed to estimate the average values of synaptic number or the summed density values of PSD width and cleft width in the synapse.

### Novel object recognition

The standard novel object recognition (NOR) task procedure reported in previous studies was followed (Ennaceur and Delacour, 1988; Wang et al., 2020a). The NOR design is shown in **Figure 6A**; it was tested in three phases: habituation, acquisition, and recognition. During the habituation trial, the animals were allowed to explore an empty arena (40 × 40 × 40 cm) for 10 min. The short habituation was aimed at reducing novelty responses to the open-field apparatus, allowing the mice to explore novel stimuli in a familiar environment. During the sample trial, the mice were placed in the arena with two sample objects (objects A and B) for a period of 5 min to allow for a criterion level of investigation and familiarization of the objects. The mice were then returned to their home cage for 5 min. Subsequently, the mice were subjected to the test trial for 5 min. They were presented with a further copy of the original object A (now familiar) and a novel object C (relative to the presentation of the original object B) for 5 min of investigation. Object exploration was defined as sniffing, touching the object with the nose, or pointing the nose toward the object from a distance shorter than 2 cm. Accordingly, the durations (time) of explorations of each object were recorded for each sample or test trial and expressed as %preference indexes (PI) (Barkus et al., 2014). Between sessions and animals, the arena and objects were cleaned with 70% ethanol to eliminate olfactory cues.

### Statistical analyses

The unpaired Student's *t*-test was performed for imaging, electron microscopy, and immunoblotting data. The interaction effect of (Genotype × Phase)/(Treatment × Phase) on behavioral data was analyzed by two-way ANOVA or the unpaired Student's *t*-test, followed by Tukey's *post-hoc* test, where applicable (GraphPad Prism 6.0, La Jolla, CA, USA). All values were presented as means ± S.E.M. A p-value of < 0.05 was considered statistically significant.

## Results

### Delayed maturation of PV-positive interneuron in adult pgc-1alpha KO mice

Given that the onset of CP plasticity can be delayed by directly preventing the maturation of GABA interneuron (Mower, 1991) and that the overexpression of BDNF can promote the maturation of GABA neurons (Huang et al., 1999), we evaluated the role of *Pgc-1alpha* in maintaining and promoting the maturation of PV-positive interneurons. In all investigated ages (preweaning at day 14 [P14] and young adult at day 100 [P100]), we first observed a remarkable reduction in the density of PV-positive neurons in *Pgc-1alpha* KO (Cre-f/f) mice compared with the age-matched control mice (Figures 1C–C', D–D', E–E'). Next, we investigated the maturation of PVI in adult brains of *Pgc-1alpha* KO mice by quantifying the number of cells expressing BDNF. Notably, *Pgc-1alpha* deletion reduces the density of BDNF-positive populations compared with the control population at day 100 (Figures 1F–F', G–G'), suggesting that *Pgc-1alpha* deletion disturbs CP plasticity, possibly by preventing the maturation of GABA interneurons.

### Increased damages of PNNs and prolonged closure of CP timing in adult pgc-1alpha KO mice

To elucidate the molecular mechanisms of low PGC-1α content with reduced density of PNNs, we first investigated the degree of oxidative stress with 8-oxo-dG, a marker for DNA oxidative damage. We observed a significantly higher 8-oxo-dG labeling in the *Pgc-1alpha* KO cortex of young adult mice at day 90 [P90] than in that of the control mice (Figures 2C–C'). The presence of well-formed PNNs around PVIs not only contributes to protecting cells against oxidative stress but also limits adult plasticity. Utilizing WFA, a N-acetylgalactosamine (GalNac)-specific plant lectin that can bind PNN components, we confirmed that WFA-labeled PNNs gradually increase to encapsulate the PVI cell body from the preweaning day [P20] to the young adult day [P90] (Figures 2B–B'). To determine whether *Pgc-1alpha* deletion alters adult plasticity, double immunofluorescent labeling for PV and PNN was performed. A significant reduction in the number of PVIs (PV^+^ cells) and PNN-enwrapped PVIs (PV^+^WFA^+^ cells) was observed in 90-day-old mice KO compared with the age-matched control mice (Figures 2D–D”, E). Furthermore, a remarkable reduction in the aggrecan level expression was also observed (Figures 2G–G'). Therefore, prolongation of the closure of CP timing by *Pgc-1alpha* deletion is mediated, at least partly, by increased oxidative damage of PNNs and molecular “brakes” during the later stage of PVI maturation.

MMP9 is a secreted endopeptidase that regulates PNN formation and organization by cleaving extracellular matrix (ECM) components (Wen et al., 2018a). To explore the mechanism by which *Pgc-1alpha* deletion induced PNN integrity damages, the MMP9 level expression was compared within the two genotypes at early adult age [P90]. A significantly higher MMP9 signal was observed in the cortex of the 90-day-old KO than in that of the age-matched control mice (Figures 2F–F', H–H'). Collectively, these results provide causal evidence that PGC-1α affecting PNN regulated the closure of the CP window not only through its antioxidant function but also by regulating MMP9 expression.

### Disturbed synaptic ultrastructure and plasticity in adult Pgc-1alpha KO mice

**Figure 3:**
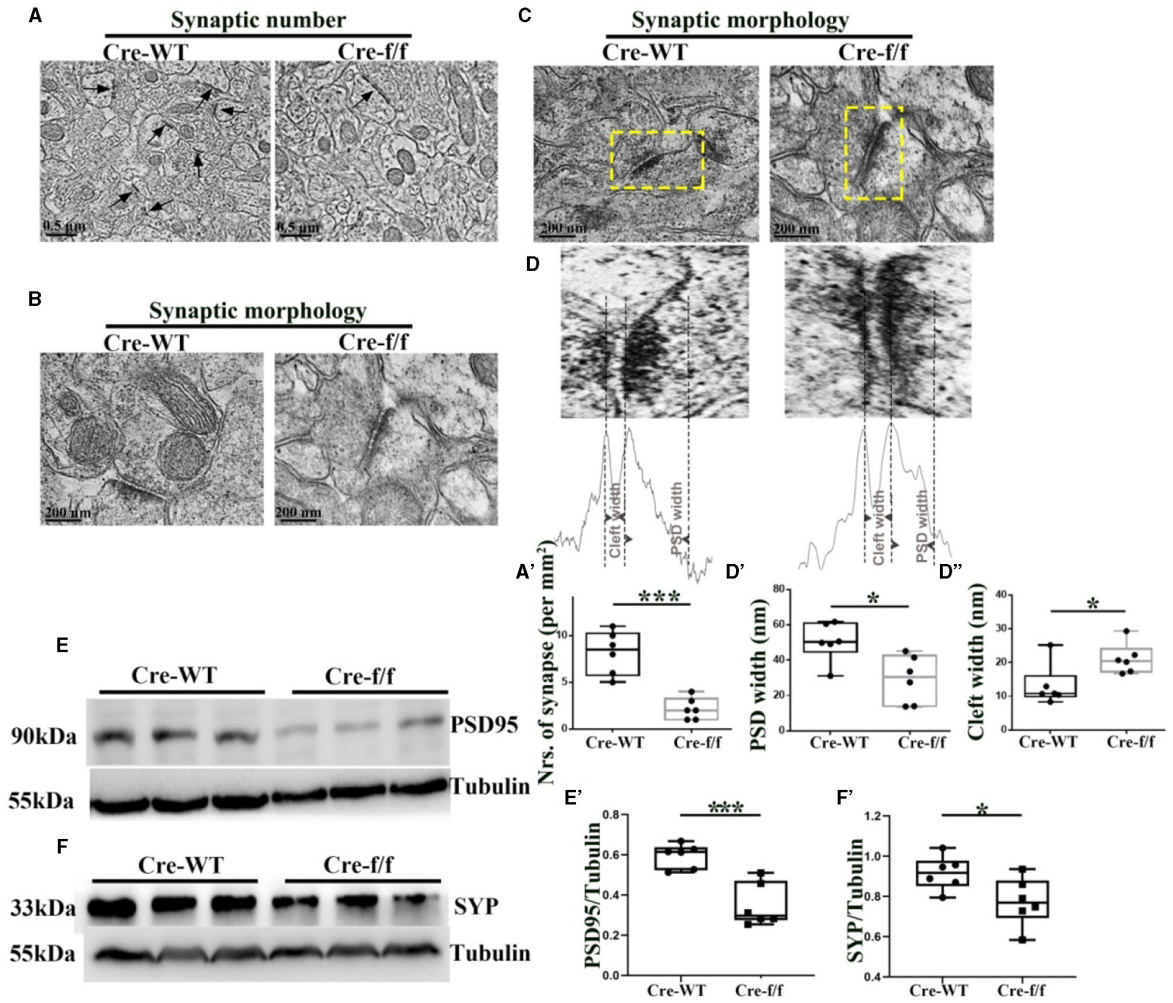
*Pgc-1alpha* deletion disturbs synaptic ultrastructure. **(A)** Low-power electron micrographs showing representative neuronal synapses sampled from the cortex of Cre-WT and Cre-f/f transgenic animals. The total number of synapses sampled from the cortex was counted during stereological measurements. Some synapses are indicated with arrows. Scale bar = 0.5 nm. **(B)** High-power electron micrographs showing the two major morphological types of synapses in the cortical neuron. Scale bar = 200 nm. **(C)** Area selected for measurements (yellow box in the upper micrograph). Scale bar = 200 nm. **(D)** Postsynaptic density (PSD) width and cleft width are indicated with arrowheads. Line graph showing summed density values of PSD width and cleft width in the synapse. The quantification of **(A')** synaptic number, **(D')** PSD width, and **(D”)** cleft width is illustrated in the box plot. The expression pattern and quantification of two synaptic biomarkers **(E–E')** PSD95 and **(F–F')** synaptophysin (SYP), were studied by western blotting in both genotypes. Values are expressed as means ± S.E.M. *n* = 6/group. Significant levels were set at *p < 0.05, and ***p < 0.001 denotes noted differences between Cre-WT and Cre-f/f animals.

To assess the effect of *Pgc-1alpha* gene deletion on synaptic plasticity, we used quantitative stereological measurements and evaluated the number of neuronal synapses from the cortex between Cre-WT and Cre-f/f transgenic animals at early adult age [P90]. A remarkable reduction in the synaptic number (Figures 3A–A') was observed in the cortex of the Cre-f/f mice compared with the Cre-WT mice. We also compared features of the synaptic ultrastructure between the two genotypes. The width of PSD and the synaptic cleft were determined by an unbiased algorithm using grayscale levels (Figures 3C, D). A remarkable decrease in the PSD width (Figures 3D, D') and a notable increase in the cleft width (Figures 3D, D”) were observed in the Cre-f/f mice compared with the Cre-WT mice. Two synaptic biomarker candidates, SYP and PSD95 were selected to complement the ultrastructural data at the light microscopic level. Compared with the control mice, a remarkable reduction in the levels of PSD95 (Figures 3E, E') and SYP (Figures 3F, F') expression was observed in the *Pgc-1alpha* KO mice, suggesting that prolonged CP timing induced by *Pgc-1alpha* gene deletion was accompanied by disturbed synaptic plasticity during cortical development.

### MMP inhibitor protects PVIs against oxidative stress in adult pgc-1alpha KO mice

**Figure 4:**
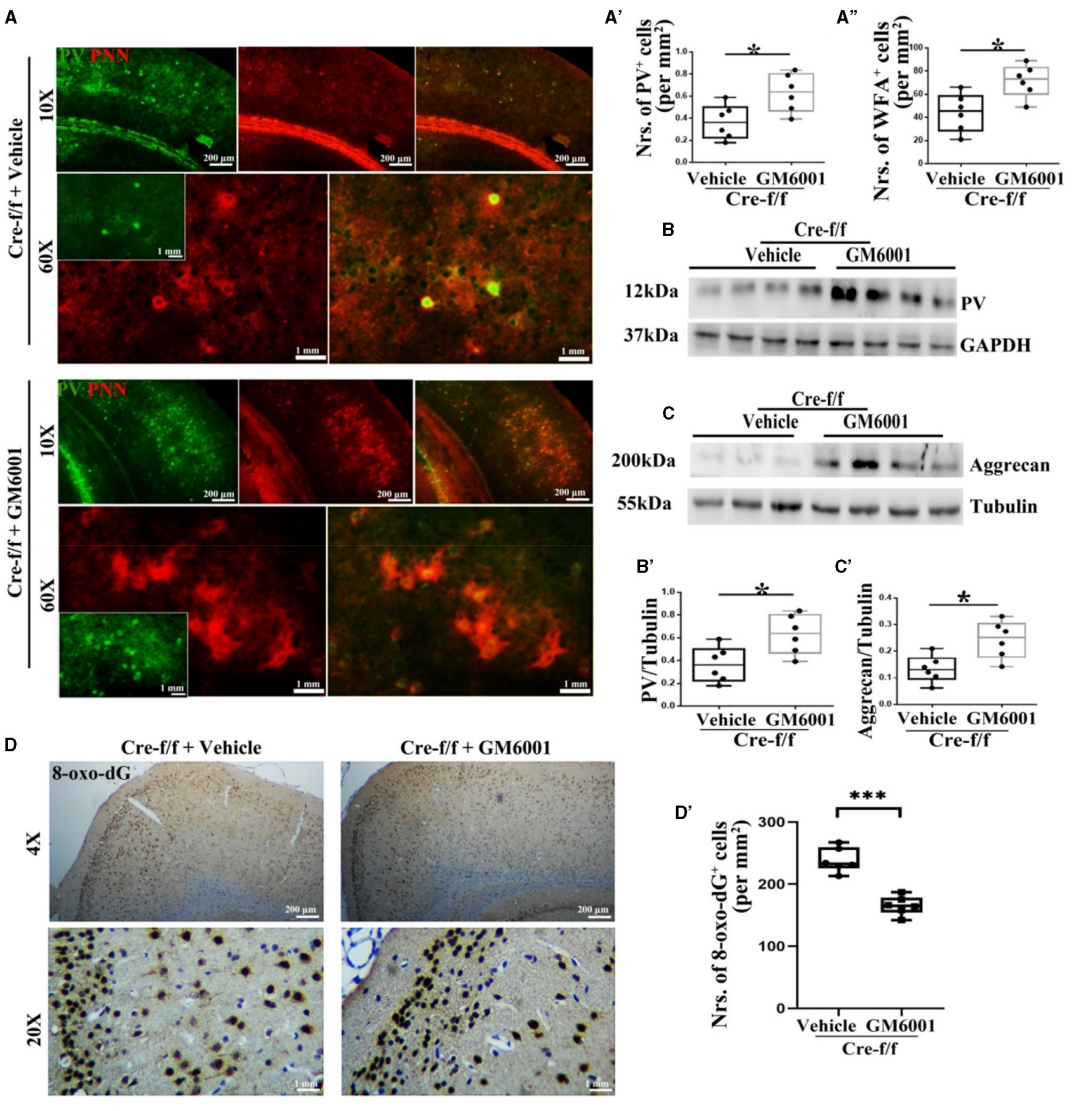
MMP inhibitor protects PVI resisting to oxidative stress by regulating their surrounding PNN in *Pgc-1alpha* KO mice. A vehicle or MMP inhibitor was administered intraperitoneally to *Pgc-1alpha* KO mice (Cre-f/f) once a day for 11 days. **(A)** Micrographs show double-labeling for PV and WFA-labeled PNNs in the RC of both treatments. Green, PV; Red, WFA-labeled PNNs. The number of **(A')** PV^+^ cells and **(A”)** WFA^+^ cells are illustrated in the box plot. For each group, *n* = 6. Values are expressed as means ± S.E.M. Cortical lyases of vehicle- or MMP inhibitor-treated *Pgc-1alpha* genotype mice were immunoblotted using antibodies against PV and aggrecan. The expression pattern and quantification of **(B, B')** PV and **(C, C')** aggrecan in the cortex of two treatments were studied by western blotting. Tubulin/GAPDH was used as the loading control. Values are expressed as means ± S.E.M. *n* = 6/group. **(D)** Immunohistochemistry using an antibody specific to 8-oxo-dG was conducted on 30-μm coronal brain sections of Cre-f/f mice in both treatments. **(D')** The quantification of 8-oxo-dG^+^ cells for immunohistochemistry in the cortex is shown in the bar graph. The unpaired Student's *t*-test was performed for imaging analysis and immunoblotting data. Values are expressed as means ± S.E.M. *n* = 6/group. Significant levels were set at *p < 0.05 and ***p < 0.001.

Based on the results that *Pgc-1alpha* deletion caused a remarkable increase in the number of cells expressing MMP9, we then investigated whether inhibiting the disruption of PNNs could protect PVIs against oxidative damage. As a broad-spectrum MMP inhibitor, GM6001 was injected intraperitoneally into *Pgc-1alpha* KO mice for 11 days (Wang and Tsirka, 2005). As shown in Figure 4, among the young adult [P90] *Pgc-1alpha* KO mice, paralleling increased levels of PV (Figures 4A–A', B–B') and WFA-labeled PNNs (Figures 4A, A”, C–C'), a decreased degree of oxidative stress (8-oxo-dG^+^ cells) was observed in the GM6001-treated compared with the vehicle-treated *Pgc-1alpha* KO mice (Figures 4D–D'). This indicated that, although *Pgc-1alpha* gene deletion renders PVIs vulnerable to oxidative stress, the inhibition of MMP9-mediated pruning of PNNs increases resistance to oxidative stress, thereby affording novel therapeutic treatment strategies for psychiatric disorders associated with PGC-1α dysregulation.

### Inhibiting the pruning of PNNs ameliorates the synaptic ultrastructure and plasticity in adult pgc-1alpha KO mice

**Figure 5:**
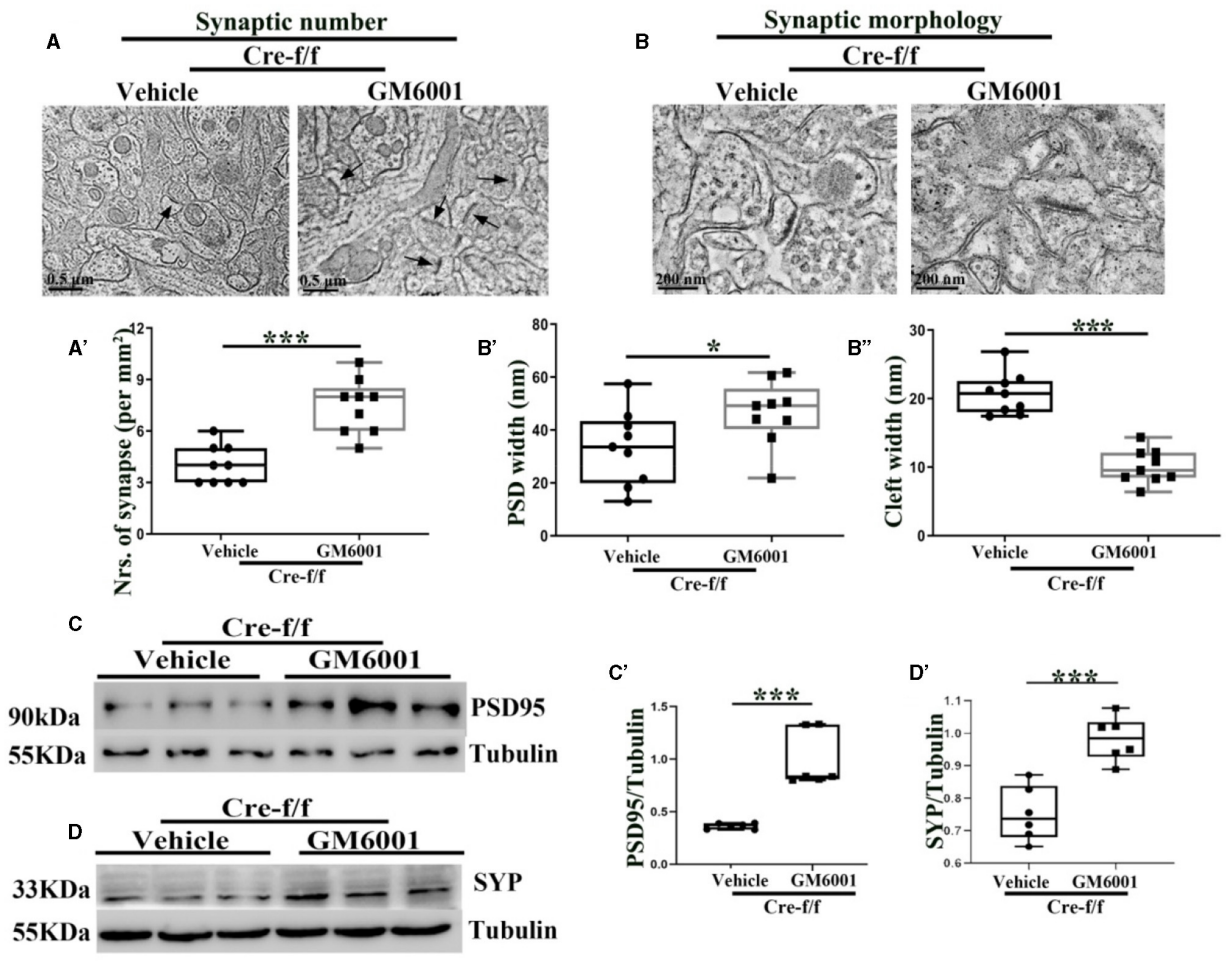
MMP inhibitor rectifies the abnormalities of the synaptic ultrastructure in *Pgc-1alpha* KO brain. A vehicle or MMP inhibitor was administered intraperitoneally to *Pgc-1alpha* KO mice (Cre-f/f) once a day for 11 days. The **(A, A')** synaptic number and **(B)** synaptic morphology, including **(B')** PSD width and **(B”)** cleft width from the cortex of the two treatments, were also investigated by electron microscopy. *n* = 6–9/group. The expression pattern and quantification of two synaptic biomarkers, **(C, C')** PSD95 and **(D, D')** SYP, were studied by western blotting in both treatments. Values are expressed as means ± S.E.M. *n* = 6–7/group. For each animal, digital images of six brain sections obtained from the cortex were analyzed to estimate the average values of the synaptic number or the summed density values of PSD width and cleft width in the synapse. The unpaired Student's *t*-test was performed for electron microscopy analysis. Significant levels were set at *p < 0.05 and ***p < 0.001.

Using ultrastructural and light microscopic approaches, we investigated whether inhibiting the pruning of PNNs could restore the abnormalities of the synaptic ultrastructure caused by *Pgc-1alpha* deletion. Compared with the vehicle-treated controls, parameters, including the synaptic number (Figures 5A–A'), PSD width (Figures 5B, B'), and the synaptic cleft width (Figures 5B, B”), were significantly altered in the GM6001-treated mice. Using the brain homogenate from the cortex of the *Pgc-1alpha* KO mice, we confirmed that GM6001 treatment increased synaptic plasticity, as demonstrated by a remarkable increase in the expression of two synaptic biomarkers, PSD95 (Figures 5C–C') and SYP (Figures 5D–D'), compared with vehicle treatment.

### Inhibiting the pruning of PNNs corrects deficits in short-term habituation and aberrant salience displayed in pgc-1alpha KO mice

**Figure 6:**
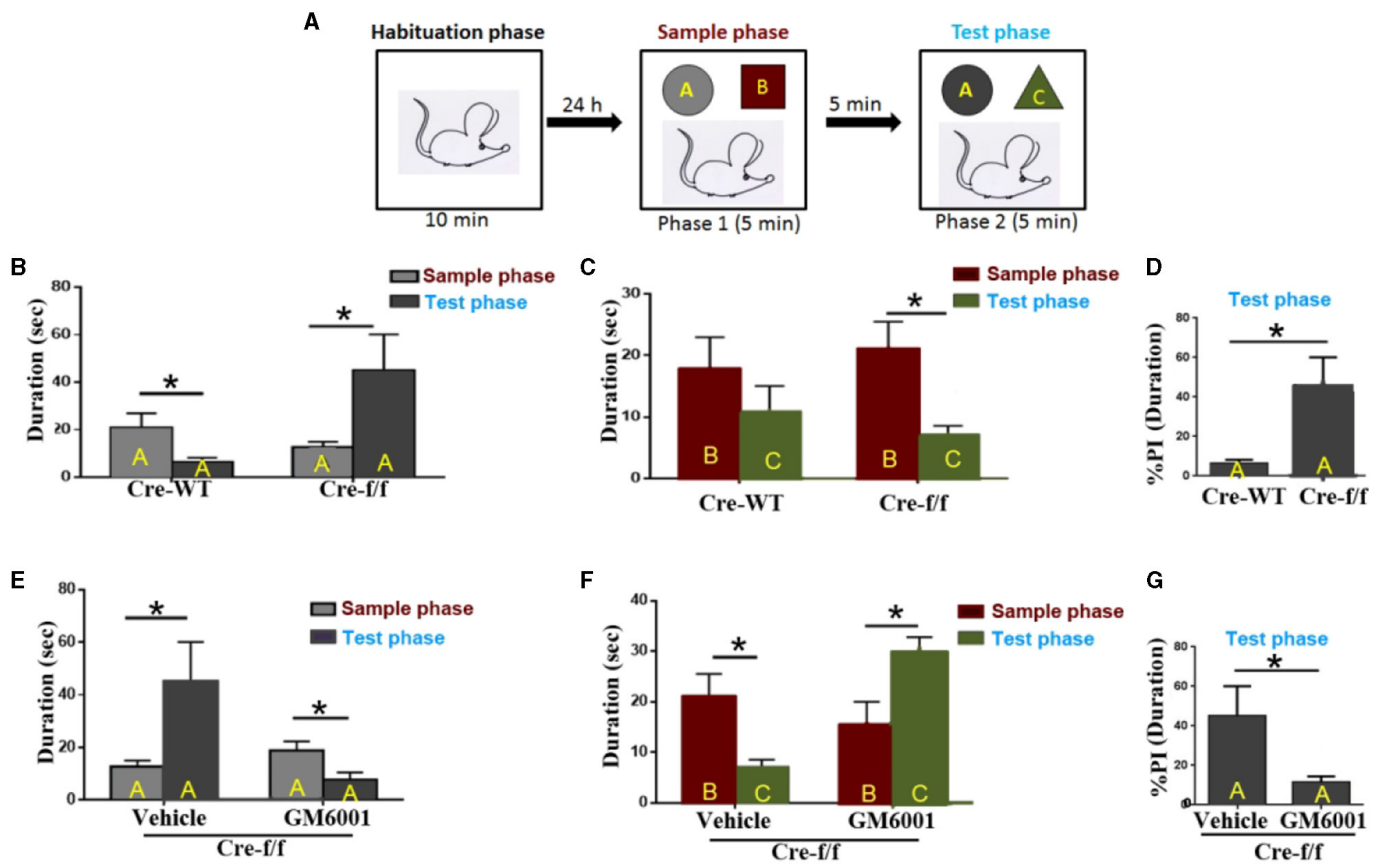
MMP inhibitor rescues short-term habituation deficits and aberrant salience displayed in *Pgc-1alpha* KO mice. **(A)** The top panel shows the design of the standard novel object recognition task. During the habituation phase, the animals were allowed to explore an empty arena for 10 min. Approximately 24 h later, the animals were exposed to two objects, **(A, B)** (sample phase), and after a 5-min interval, they received a 5-min test, in which they were allowed to explore a duplicate of the familiar object A and a novel object **(C)** (test phase). **(B)** Cre-WT (*n* = 8) and Cre-f/f (*n* = 8) mice were used to evaluate the effect of *Pgc-1alpha* deletion on short-term habituation. The time spent exploring object A between the two phases is shown in the bar graph. **(C)** The time spent exploring object B during the sample phase and object C during the test phase is shown in the bar graph. **(D)** The duration (*D*) spent exploring object A between the two genotype mice during the test phase is represented as the %preference index (PI). The PI allows discrimination between the familiar object A (*D*_A_) and the novel object C (*D*_C_) during the test phase [%PI_*A*_ = (*D*_A_)/(*D*_A_ + *D*_C_) × 100] for each genotype mice. **(E)** Cre-f/f mice treated with the vehicle (n = 8) or GM6001 (n = 8) were used to evaluate the effect of the MMP inhibitor on short-term habituation deficits and aberrant salience in *Pgc-1alpha* KO mice. The time spent exploring object A between the two phases is illustrated in the bar graph. **(F)** The time spent exploring object B during the sample phase and object C during the test phase is shown in the bar graph. **(G)** The duration spent exploring object A between the two treatments during the test phase is represented as the %PI. Behavioral data were analyzed by two-way ANOVA or by performing the unpaired Student's *t*-test, followed by Tukey's *post-hoc* test, where applicable. All values are presented as means ± S.E.M. Significant levels were set at *p < 0.05.

By performing the NOR test, we reproduced our previous finding that *Pgc-1alpha* deletion displays short-term habituation deficit and aberrant salience (Figures 6A–D). Specifically, compared with the control mice, the *Pgc-1alpha* KO mice paid more attention to a further copy of the original object A (now familiar), relative to the presentation of the novel object C (Figure 6D), during the test phase (Wang et al., 2020a).

We then compared the treatment (vehicle/GM6001) effect on the behavioral abnormalities displayed in the *Pgc-1alpha* KO (Cre-f/f) mice. As can be observed in Figure 6E, the vehicle-treated Cre-f/f mice showed a preference for object A when the test phase (phase 2) was compared with the sample phase (phase 1), but the GM6001-treated Cre-f/f mice showed less preference for object A when phase 2 was compared with phase 1. Figure 6F presents the difference in duration for object B during the sample phase (phase 1) and object C during the test phase (phase 2) for Cre-f/f mice with the two treatments. As can be observed, different from the vehicle-treated, the GM6001-treated Cre-f/f mice showed a significant increase in exploring preference for object C during phase 2 compared with object B during phase 1, indicating that the MMP inhibitor rescued the pronounced deficit in short-term habituation exhibited in *Pgc-1alpha* KO mice. Figure 6G shows the difference in % duration for object A calculated using the formula [(time at object A)/(time at object A + time at object C)] in the two treatments. Specifically, compared with the vehicle-treated, the GM6001-treated Cre-f/f mice paid less attention to a further copy of the original object A (now familiar), relative to the presentation of the novel object C, during the test phase (Figure 6G).

## Discussion

In general, SZ is heritable, and several genes are associated with the disease. Recently, the Christoforou's laboratory focused on chromosome 4p15-p16, a well-established candidate region for SZ, and identified that *Pgc-1alpha* is one of the top candidate genes of SZ (Christoforou et al., 2007). Based on this report, we generated *Pgc-1alpha* KO mice and confirmed that these mice presented some characteristic features of SZ, such as hyperactivity, reduced prepulse inhibition, and exaggerated startle reactivity (Wang et al., 2019, 2020a). Although *Pgc-1alpha* deficiency results in SZ-like behavioral abnormalities, the detailed mechanisms remain largely unknown. Considering the pivotal role of PVI maturation in both CP opening and closure (by their surrounding PNNs), on the one hand, and their impairment in SZ patients and animal models, on the other hand (Cardis et al., 2018), we investigated the role of PGC-1α in regulating PVI-mediated CP opening and closure. In line with the report that overexpressing PGC-1α robustly induced the expression of PV in neuroblastoma cells, in this study, we confirmed that behavioral dysfunction exhibited in *Pgc-1alpha* KO mice is accompanied by a reduced number of PVIs. Utilizing an unbiased gene array on neuroblastoma cells, Lucas et al. investigated downstream gene targets of PGC-1α in the brain and discovered that PGC-1α overexpression upregulated developmentally relevant genes, including synaptotagmin 2 (syt2), complexin 1 (cplx1), and neurofilament heavy chain (Nefh) expressed in cortical interneuron (Lucas et al., 2014). Consistent with this finding, the conditional deletion of *Pgc-1alpha* in PV-positive neurons significantly decreased the cortical transcript expression of these genes (Lucas et al., 2014), suggesting that PGC-1α is required for the developmental induction of these genes in PV-positive interneurons.

Neuronal circuits in the brain are shaped by the experience during CP in early postnatal life; this activity-dependent development is triggered by the functional maturation of local inhibitory connections and is driven by PVI large basket cells at different rates across brain regions (Hensch, 2005). The activity-dependent expression of BDNF regulates the maturation of PVI and, consequently, the duration of the postnatal CP for experience-dependent plasticity. Being one of the vital extrinsic factors, BDNF, which appears ahead of CP onset, contributes to the health and maintenance of PVIs. A decrease in BDNF protein levels observed in *Pgc-1alpha* gene deletion mice thus suggests that the vulnerability of PVI is increased in these animals. Increased expression of BDNF in the brain can be induced by a PGC-1α/FNDC5 pathway during endurance exercise (Wrann et al., 2013). However, further studies are necessary to clarify whether the decrease in PV protein expression in *Pgc-1alpha* gene deletion mice is due to a decrease in BDNF expression.

Emerging evidence indicates that PNNs, covering the soma and proximal neuritis of the subpopulation of neurons (Giamanco et al., 2010), were markedly altered in subjects with SZ (Berretta et al., 2015). Long believed to be responsible primarily for structural support and neuronal protection against oxidative stress, PNNs have gradually come to the forefront of neuroscience research as critical elements that regulate synaptic plasticity during development (Berretta et al., 2015). A juvenile form of PNNs, supportive of neurogenesis, axonal outgrowth, and synaptogenesis, is substituted by an adult form, which predominantly restricts plasticity. The implementation of this adult form of PNNs coincides with CP closure during postnatal development, during which time neuronal circuits are shaped, and culminates with the maturation of PNNs (Berretta et al., 2015). Converging evidence indicates that prolonged CP timing is consistent with a delayed or extended period of circuit instability (Wen et al., 2018b). Various social and intellectual cognitions, which should be consolidated during CPs, thus seem to remain open to fluctuations in adulthood (Wang et al., 2020b). The influence of *Pgc-1alpha* deletion-induced oxidative stress was reported in our previous study (Wang et al., 2020b); however, whether *Pgc-1alpha* deletion can affect MMP9 expression has not been investigated to date. Both the increased degree of oxidative stress and the elevated number of cells expressing MMP9 contribute to the vulnerability of PNN damage in the *Pgc-1alpha* KO brain. These results suggest that *Pgc-1alpha* deletion potentially affects cortical plasticity due to a failure in the closure of the CP window.

Astrocytic PGC-1α has been reported to play a role in the maturation of astrocytes and the formation and function of neighboring synapses (Zehnder et al., 2021), while neuronal PGC-1α has been reported to be essential for the maintenance of dendritic spines (Cheng et al., 2012). We then investigated the role of PGC-1α restricted to GABAergic neurons. Given the previous description that deletion of the *Pgc-1alpha* gene is accompanied by an obvious disturbance of PVI-involved gamma oscillations (Brady et al., 2016), one might speculate that the *Pgc-1alpha* gene plays a pivotal role in regulating the synaptic connectivity of neuronal circuits in the brain's cortex. This should potentially result in corresponding synaptic changes, including reduced synaptic number and reduced receptor content (Posfai et al., 2016). As expected, the present results demonstrated that *Pgc-1alpha* deletion induces morphological changes in synapses. Specifically, the synaptic number in the cortex of the *Pgc-1alpha* KO mice was significantly reduced when compared with control littermates, and the diminished population of synapses was characterized by increased cleft width and decreased PSD width. These results are consistent with the report that PGC-1α is required for a developmental gene program regulating syt2 and Cplx, two high-affinity Ca^2+^ sensors essential for synchronous neurotransmitter release, as well as the structural protein Nefh, which is an excellent marker for analyzing the development and plasticity of inhibitory neurons in the cortex (Yamauchi et al., 2005; Sommeijer and Levelt, 2012; Lucas et al., 2014; Park et al., 2016). Additionally, considering the role that PNNs play in physically preventing neurite pruning and outgrowth (Do et al., 2015), we suggest that changes in plasticity during adult cortical development are triggered by Pgc-1alpha deletion, at least partly due to the loss of PNNs, the structural obstacles that limit plasticity in adults.

Given the role of PNN in regulating the CP plasticity window, the inhibition of PNN pruning is needed for future research to correct circuit instability. Compelling evidence supports the frequent occurrence of impaired PNNs with enhanced activity of MMP9, one of the ECM-modifying enzymes observed in post-mortem brain tissues of patients with SZ (Beurdeley et al., 2012). A functional polymorphism of the *MMP9* gene (C(-1562)T) was reported in SZ patients (Rybakowski, 2009). These findings, along with the finding that the activation of peroxisome proliferator-activated factor receptor-γ (PPAR-γ) could inhibit MMP9 activity (Lee et al., 2004), has led us to suspect that PGC-1α, the coactivator of PPAR-γ, could regulate MMP9 expression. As expected, upon paralyzing less-condensed PNNs, an elevated MMP9 level was observed in the *Pgc-1alpha* KO mouse brain. In light of that, strategically introduced interventions to block PNN damage may be a novel preventive therapy for psychiatric disorders associated with PGC-1α dysregulation.

GM6001, a broad-spectrum MMP inhibitor, has been reported to provide neuroprotection with respect to both morphology and neurological function in the immature brain of a rat model following hypoxia-ischemia (Chen et al., 2009). In this study, we observed that GM6001 ameliorates short-term habituation deficit and aberrant salience displayed in *Pgc-1alpha* KO mice. Given the description that fast-spiking PVIs are inhibitory interneurons implicated in shaping excitatory/inhibitor (E/I) balance and network oscillatory activity, we suggest that the amelioration effect of GM6001 on SZ-like behavioral abnormalities of *Pgc-1alpha* KO mice is mediated, at least partly, by the alteration of the role of PVI in the regulation of CP opening and closure (by their surrounding PNNs). It has been reported that microglia are currently considered a source of MMPs in psychiatric disorders, thus being responsible for PNN manipulation (Crapser et al., 2020; Wegrzyn et al., 2021). PGC-1α downregulation suppresses inflammation in the substantia nigra of PD mice by inhibiting microglia activity (Guan et al., 2023). However, further studies are necessary to clarify whether the impact of PNN integrity and synaptic plasticity by PGC-1α is due to an alteration of microglia activity.

## Conclusion

In this study, we demonstrated that the effect of PGC-1α on cortical plasticity is mediated, at least partially, by the regulation of CP onset/closure. Strategically introduced reinforcement of molecular brakes may offer a novel preventive therapy for psychiatric disorders associated with PGC-1α dysregulation.

## Data availability statement

The original contributions presented in the study are included in the article/supplementary material, further inquiries can be directed to the corresponding authors.

## Ethics statement

The animal studies were approved by Jiangsu University (SYXK2018-0053), and the Chinese Council on Animal Care and the National Institutes of Health Guide for the Care and Use of Laboratory Animals (NIH Publications No. 8023, revised 1978). The studies were conducted in accordance with the local legislation and institutional requirements. Written informed consent was obtained from the owners for the participation of their animals in this study.

## Author contributions

JW and W-NZ were involved in resource collection and conceptualized the study. W-JZ, H-ZS, M-NG, L-FX, H-RZ, Z-ZL, and Y-QZ were involved in the methodology. JW, W-JZ, and H-ZS wrote the original draft. JW, W-JZ, and W-NZ reviewed and edited the manuscript. All authors contributed to the article and approved the submitted version.
